# Community succession and functional prediction of microbial consortium with straw degradation during subculture at low temperature

**DOI:** 10.1038/s41598-022-23507-z

**Published:** 2022-11-23

**Authors:** Xin Zhang, Qinggeer Borjigin, Ju-Lin Gao, Xiao-Fang Yu, Shu-Ping Hu, Bi-Zhou Zhang, Sheng-Cai Han

**Affiliations:** 1 Agricultural College, Inner Mongolia Agricultural University, No. 275, XinJian East Street, Hohhot, 010019 China; 2 Life Sciences College, TongHua Normal University, No. 950, YuCai Road, Tonghua, 314002 China; 3 Key Laboratory of Crop Cultivation and Genetic Improvement in Inner Mongolia Autonomous Region, No. 275, XinJian East Street, Hohhot, 010019 China; 4 Hortlculture and Plant Protection College, Inner Mongolia Agricultural University, No. 29, Eerduosi East Street, Hohhot, 010019 China; 5 Vocational and Technical College, Inner Mongolia Agricultural University, Altan Street, Baotou, 014109 China; 6 Special Crops Institute, Inner Mongolia Academy of Agricultural Animal Husbandry Sciences, No. 22, ZhaoJun Road, Hohhot, 010031 China

**Keywords:** Biological techniques, Biotechnology, Ecology, Microbiology, Molecular biology

## Abstract

To systematically explore and analyze the microbial composition and function of microbial consortium M44 with straw degradation in the process of subculture at low temperature. In this study, straw degradation characteristics of samples in different culture stages were determined. MiSeq high-throughput sequencing technology was used to analyze the evolution of community structure and its relationship with degradation characteristics of microbial consortium in different culture periods, and the PICRUSt function prediction analysis was performed. The results showed that straw degradation rate, endoglucanase activity, and filter paper enzyme activity of M44 generally decreased with increasing culture algebra. The activities of xylanase, laccase, and lignin peroxidase, as well as VFA content, showing a single-peak curve change with first an increase and then decrease. In the process of subculture, Proteobacteria, Bacteroidetes, and Firmicutes were dominant in different culture stages. *Pseudomonas*, *Flavobacterium*, *Devosia*, *Brevundimonas*, *Trichococcus*, *Acinetobacter*, *Dysgonomonas*, and *Rhizobium* were functional bacteria in different culture stages. It was found by PICRUSt function prediction that the functions were concentrated in amino acid transport and metabolism, carbohydrate transship and metabolism related genes, which may contain a large number of fibers and lignin degrading enzyme genes. In this study, the microbial community succession and the gene function in different culture periods were clarified and provide a theoretical basis for screening and rational utilization of microbial consortia.

## Introduction

Lignocellulose is one of the most abundant renewable carbon sources in the biosphere, and its resource utilization efficiency has potential significance for sustainable development and environmental protection^1,2^. However, inefficient lignocellulose deconstruction is a primary bottleneck for its economic conversion and further utilization (i.e., of hemicellulose and cellulose, which are enclosed by lignin)^3,4^, especially under low-temperature conditions. Biodegradation, accomplished through coordination of various microorganisms, is currently considered a highly efficient method for lignocellulosic degradation^5,6^. Previous studies have shown that more efficient and suitable strains can be screened from similar ecological environments according to application purposes^7^. And complete degradation of lignocellulose requires the synergistic action of various microorganisms in the natural environment^8–10^. Zheng et al.^11^ obtained the lignocellulose-degrading bacterium LTF-27 from cold perennial forest soil, which mainly composed of *Parabacteroides*, *Alcaligenes*, *Lysinibacillus*, *Sphingobacterium*, and *Clostridium*. Alessi et al.^12^ showed that *Asticcacaulis*, *Leadbetterella*, and *Truepera* played a key role in wheat straw degradation. Wang et al.'s^13^ study showed that a lignin-degrading composite bacteria LDC was obtained from the root soil of rotten reed straw by restricted enrichment culture method, *Pseudomonas*, *Pannonibacter*, *Thauera*, *Ruminofilibacter* and *Anaerocolumna* were main bacteria that have played an important role in the process of corn straw degradation^14^.

To promote the in situ return of corn straw to the field in the low-temperature growing area in northern of China, the dried dung from the low temperature (−7 to 8 ℃) ecological environment was used as the original material for screening corn straw degradation microbial consortium at low temperature by our research team. Finally, a microbial consortium M44 was obtained by low temperature restriction subculture, and the straw degradation rate in laboratory was more than 30%^15^. However, the correlation of species composition and the succession rule of microbial community of the M44 in different culture periods are not clear. Therefore, in this study, original samples and series of bacteria in different subculture periods of the screened microbial consortium M44 were used as materials to measure its degradation characteristics and the composition and relative abundance of microbial classification units in the process of subculture at low temperature were analyzed by using 16S rRNA gene amplification method. And the relationship between the microbial community composition and the degradation characteristics of the M44 in different culture stages was revealed, and the gene function was explored preliminary.

## Materials and methods

### Experiment materials

Air-dried sheep dung (S) was taken from Chenbarhu Banner, Hulunbuir City, Inner Mongolia, China (125°07′ E, 46°28′ N). The tested straw degradation microbial consortium M44 was screened by our laboratory^15^.

Corn straw was taken from the experimental field of the Corn Center of Inner Mongolia Agricultural University (110°28′ E, 40°32′ N), and the cellulose, hemicellulose and lignin contents were determined to be 47.23%, 34.34%, and 16.77%, respectively. Corn straw of moderate thickness and with no pests or disease was selected, washed and dried (60 °C), and cut into small pieces of 2–3 cm for use.

### Medium and culture conditions

Mandels medium (M medium) was composed of K_2_HPO_4_ (3.0 g), NaNO_3_ (3.0 g), CaCl_2_ (0.5 g), MgSO_4_·7H_2_O (0.5 g), Fe_2_SO_4_·7H2O (7.5 mg), MnSO_4_·H_2_O (2.5 mg), ZnSO_4_ (2.0 mg), CoCl_2_ (3.0 mg), and distilled water (1 L). Then**,** 40 mL M medium and 1.0 g corn straw were added to a 100-mL triangular flask for subsequent subculture, and the corn straw degradation ratio and enzyme activity were measured. Following this, the mixtures were sterilized at 121 °C for 20 min and set aside ^15^.

### Cultivation of microbial consortium

2 g dried dung was put into a triangular bottle filled with 40 mL sterile distilled water and glass beads and placed on a shaker at 15 °C for 2 h. Then, 5% (V/V) supernatant was absorbed in 40 mL M medium, corn straw was used as the substrate carbon source, and cultured at 15 °C for 21 days. After culturing for 21 days, the fermentation liquid at an inoculation rate of 5% (V/V) was transferred to new M medium and cultured successively until the 11th generation (F11). Among them, the F1, F5, F8, and F11 generations of M44 were stored at –80 °C for later use.

### Determination of straw degradation characteristics

The fermentation broth of microbial consortium M44 at F1, F5, F8, and F11 generations was inoculated into 40 mL M medium at 5% (V/V) and cultured at 15 °C for 21 days. Then, the corn straw degradation ratio was determined using the weight loss method. 5 mL of fermentation material was centrifuged at 12,000 rpm at 4 °C for 10 min and the supernatants were used as extracellular crude enzyme samples to analyze the enzyme activities and volatile fatty acid (VFA) content in F1, F5, F8, and F11 generations. Filter paper enzyme activity and endonuclease 1,4-β-glucanase activity were assessed using the DNS method^15^, and xylanase activity was established by DNS method^16^. The activity of laccase was assessed using the ABTS method, and that of lignin peroxidase was examined according to the resveratrol method^17^. One milliliter supernatant was integrated into a 1.5-mL centrifuge tube, and an acid adsorbent was added to an Agilent GC-6890 N meteorological chromatographic column was an Agilent l9091F-112 (length 30 m, inner diameter 0.32 mm, film thickness 0.5 µm). The column temperature was raised gradually. The gasification chamber temperature was 220 °C, the detector temperature was 240 °C, the carrier gas was hydrogen, the flow rate was 30.0 mL min^–1^, and the tail was blowing at 30 mL min^−1^ injection volumes 1 µL.

### Microbial composition analysis of S, F1, F5, F8 and F11 generations

Under aseptic conditions, genomic DNA from the original sample(S) and different culture periods (F1, F5, F8, F11) of M44 was extracted using a bacterial genomic DNA extraction kit (China, Tiengen Biochemical Technology Co., Ltd.), and a 1% agarose gel was used for electrophoresis. A NanoDrop 2000 UV-V spectrophotometer (Thermo Fisher Scientific, Waltham, MA, USA) was used to determine the concentration and purity of DNA. The hypervariable region V3–V5 of the bacterial 16S rRNA gene was amplified using primer pair 338F (5'-ACTCCTACGGGAGCAG-3') and 806R (5'-GGACTACHVGGGTWTCTAAT-3') with an ABI Gene Amp^®^ 9700 PCR thermonuclear system (Applied Biosystems, Thermo Fischer Scientific)^18^. PCR amplification system: TransStart Fastpfu DNA Polymerase, 20 μL reaction system, including 4 μL 5× Fast Pfu Buffer, 2 μL 2.5 mmol dNTPs, 0.8 μL Forward Primer(5 μmol/L), 0.8 μL Reverse Primer (5 μmol/L), 0.4 μL FastPfu Polymerase, 0.2 μL BSA, 10 ng Template DNA, supplement ddH2O to 20 μL. PCR reaction parameters: (a) 1× (3 min at 95 ℃); (b) recurring number × (30 s at 95 ℃); 30 s at 55 ℃; 45 s at 72 ℃); (c)10 min at 72 ℃, 10 ℃ until halted by user. PCR amplification products were sent to Shanghai Meiji Biomedical Technology Co., Ltd. for sequencing.

### PICRUSt function predictive analysis

In order to explore the function of dominant microbial community in straw degradation, PICRUSt was used to predict the metagenomic function composition of 16S RNA amplicon database^19^. In this method, the 16S RNA ampland database obtained by high-throughput sequencing was compared with the Greengenes database to obtain the functional information of OUT corresponding species. The functional composition of colonies was predicted based on the latest Kyoto Encyclopedia of Genes and Genomes (KEGG) database information.

### Data processing

Data on straw degradation characteristics were analyzed in IBM SPSS Statistics 25.0 (IBM Inc., Armonk, NY, USA, https://www.ibm.com/cn-zh/analytics/spss-statistics-software), R (V3.6.1) (https://www.r-project.org/) and Origin 2018 (https://www.Originlab.com) were used to create figures.

## Results and analysis

### Changes of straw degradation characteristics at different culture stages

#### Corn straw degradation ratio

**Figure 1:**
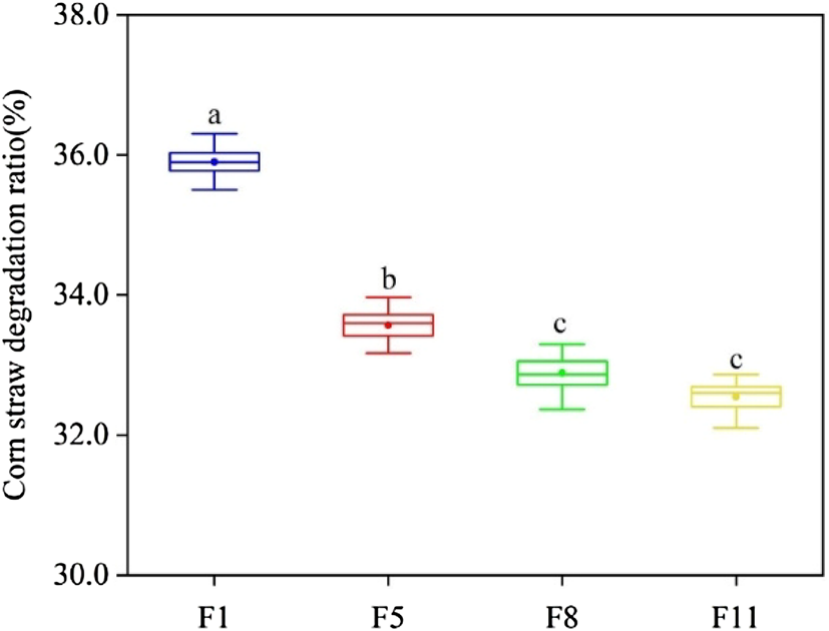
Corn straw degradation ratio was measured at different culture stages. The same small letter means there was no significant difference, and different small letters indicate significant differences at *p* < 0.05. The same follow.

Corn straw weight loss in M44 at F1 reached 35.90% at 15 ℃ for 21 days, which was greater than that at F5, F8, and F11 by 2.33%, 3.01%, and 3.35%, respectively. There were no significant differences between F8 and F11(Fig. 1).

#### Enzyme activities

**Figure 2:**
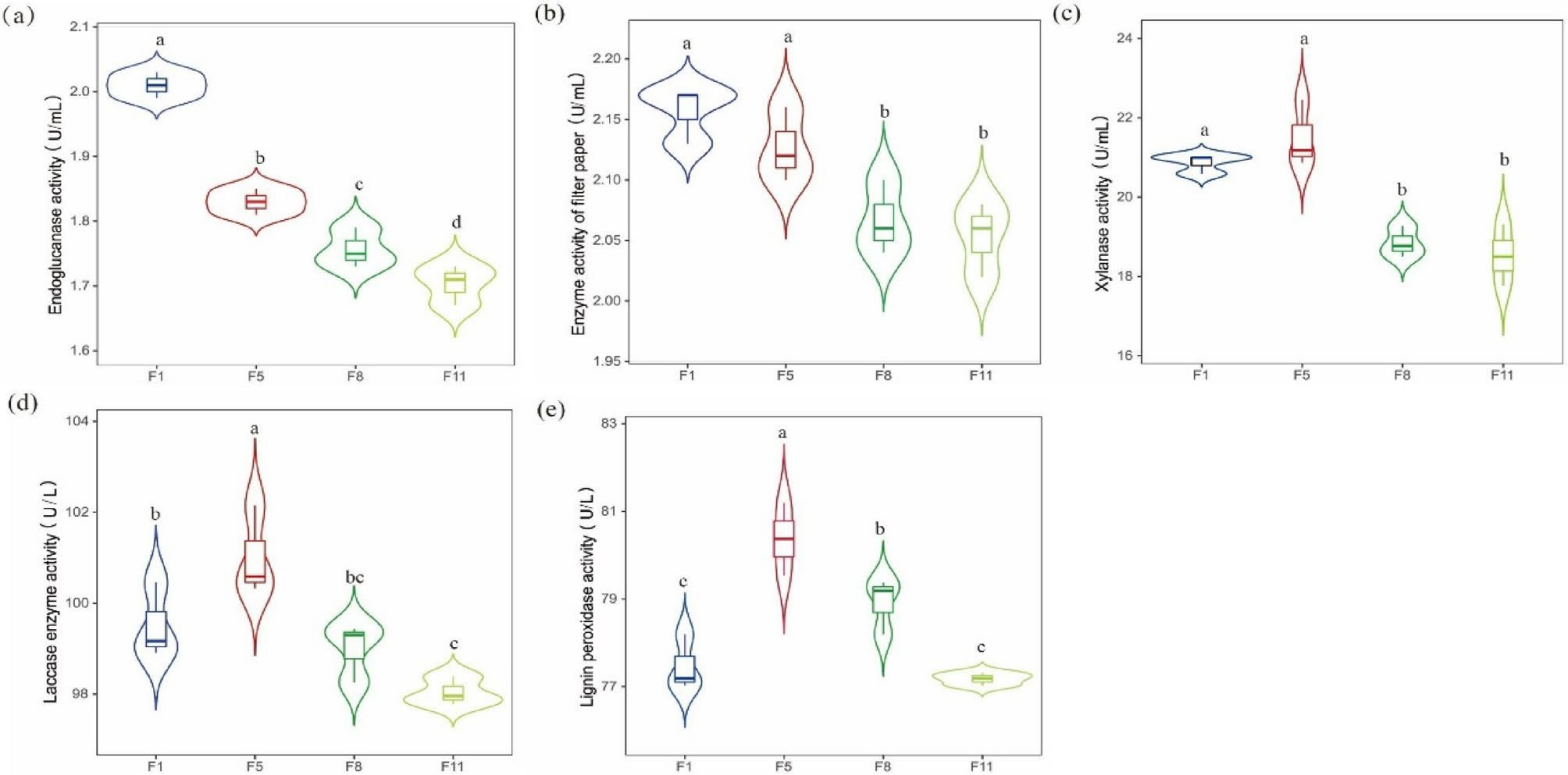
The (**a**) endoglucanase, (**b**) the filter paper enzyme, (**c**) xylanase, (**d**) laccase and (**e**) lignin peroxidase activity was measured at different culture stages.

The highest endoglucanase and filter paper enzyme activities were 2.01 and 2.16 U mL^−1^ in F1, respectively, which was significantly higher than that of F8 and F11(Fig. 2a,b). Xylanase activities was the highest in F5, with enzyme activities of 21.50 U mL^−1^, and the enzyme activities of F1 and F5 were significantly higher than those of F8 and F11(Fig. 2c). Laccase and lignin peroxidase activity reached 101.02 and 80.37 U L^−1^ at F5, which was greatly different than that of other algebras (Fig. 2d,e).

#### VFA content

**Figure 3:**
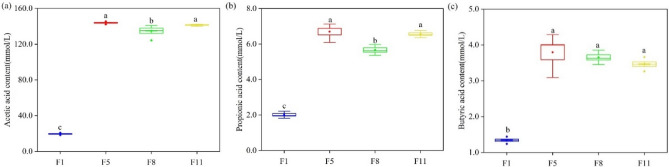
The (**a**) acetic acid, (**b**) propionic acid and (**c**) butyric acid content was measured at different culture stages.

Acetic acid, prophetic acid, and butyric acid contents were all the highest at F5. Acetic acid and propionic acid contents were 143.91 mmol L^−1^ and 6.70 mmol L^−1^ at F5, respectively, which were significantly different from those at F1 and F8 (Fig. 3a,b). Butyric acid was 3.80 mmol L^−1^ in F5, which was significantly different from that in F1 but not from that in F8 and F11(Fig. 3c).

### Alpha diversity of microorganisms at different culture stages

**Figure 4:**
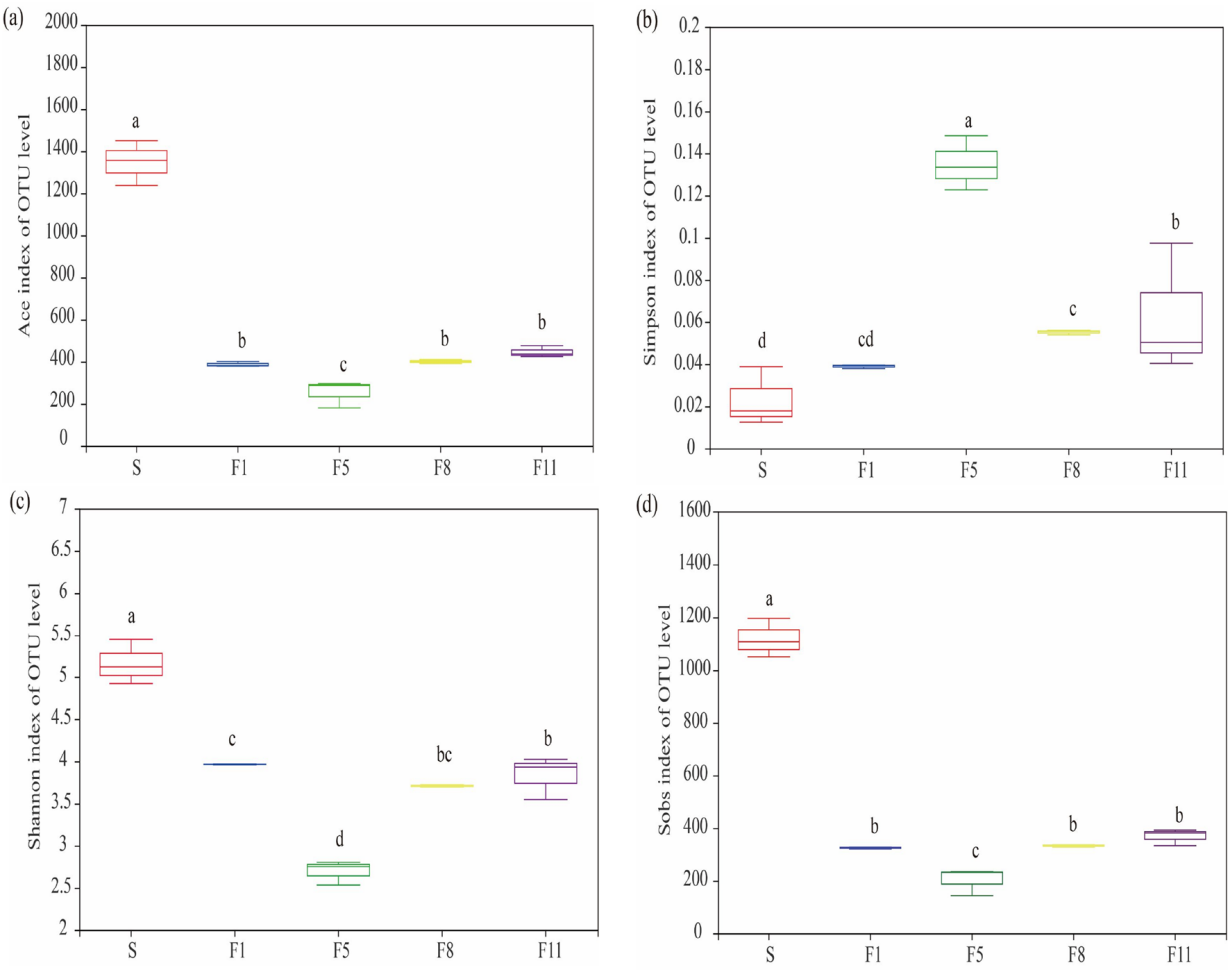
The (**a**) Ace index, (**b**) Simpson index, (**c**) Shannon index and (**d**) Sobs index in OUT level at different culture stages.

Alpha diversity was used as a measure of microbial community diversity within the sample. The Ace and Sobs indexes for the original samples(S) were 1358.22 and 1101.33, respectively, which were significantly higher than those of F1, F5, F8, F11. Shannon and Simpson indexes showed the opposite trend, with values of 2.59 and 0.161 in the F5 generation, respectively, indicating that the microbial community was more abundant and diverse in this culture stage (Fig. 4).

### Beta diversity of microorganisms at different culture stages

**Figure 5:**
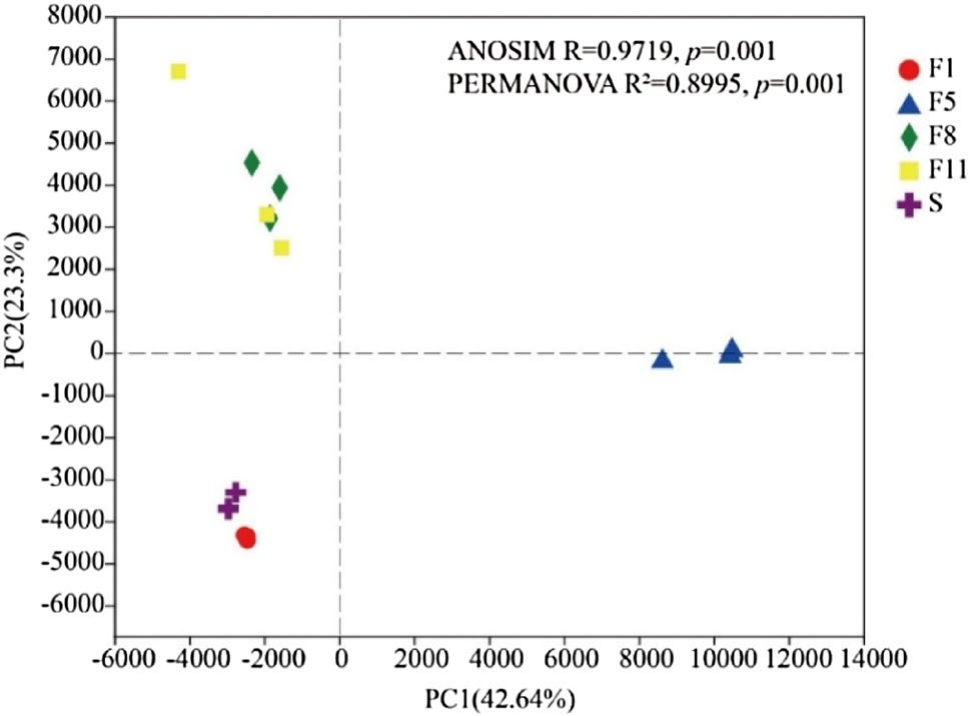
PCA map at different culture stages.

A principal component analysis (PCA) was conducted on the bacterial community in each group of samples, and the results was shown in Fig. 5. The contribution rates of PC1 and PC2 were 42.64% and 23.3% of the total, respectively. Samples of S and F1, F8, and F11 clustered together, indicating that the composition of microbial communities in these two groups was similar. On the other hand, samples from F5 clustered far from each other and into a single cluster, indicating that the microbial community compositions of the F5 samples were significantly different from those of other periods. To further define the differences, ANOSIM and PERMANOVA were performed at the OTU level based on the Bray–Curtis distance algorithm. The results showed that there were significant differences between different stages (*p* < 0.05; N = 999 permutations).

### Taxonomic composition analyses at different culture stages

#### Phylum level

**Figure 6:**
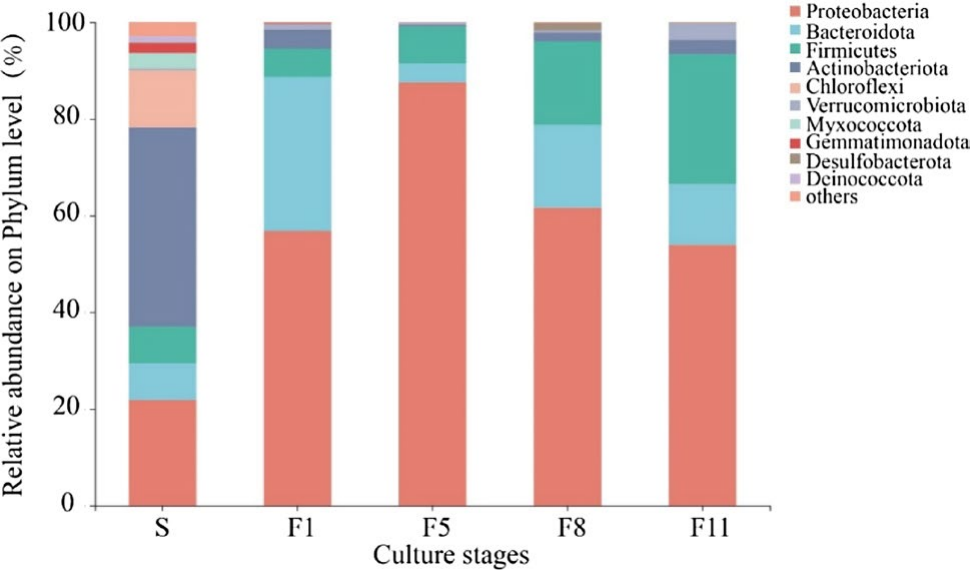
Community composition and relative abundance of bacteria at the phylum level at different culture stages. Abundances of taxa less than 1% were classified as other.

The relative abundance of bacterial groups according to classification level was shown in Fig. 6. At the phylum level, microbial consortium M44 was mainly composed of Proteobacteria, Bacteroidetes, Firmicutes, Actinobacteria, and Verrucomicrobia. Among these, Proteobacteria was dominant in M44, with abundances of 56.84%, 87.09%, 61.64%, and 53.94% at F1, F5, F8, and F11, respectively. Bacteroidota accounted for 32.11% of the total bacterial content in the F1, which was significantly higher than that of S, F5, F8, F11. The relative abundance of Firmicutes increased steadily, accounting for 26.80% of the total in F11, which was considerably higher than that at F1 (5.83%), F5 (7.68%), and F8 (17.37%). The relative abundance of Actinobacteriota in the original sample(S) was 41.19%, but decreased with subculture of the microbial consortium. The relative abundance of Verrucomicrobiota fluctuated with increasing culture stage, increasing to 3.03% in F11.

#### Genus level

**Figure 7:**
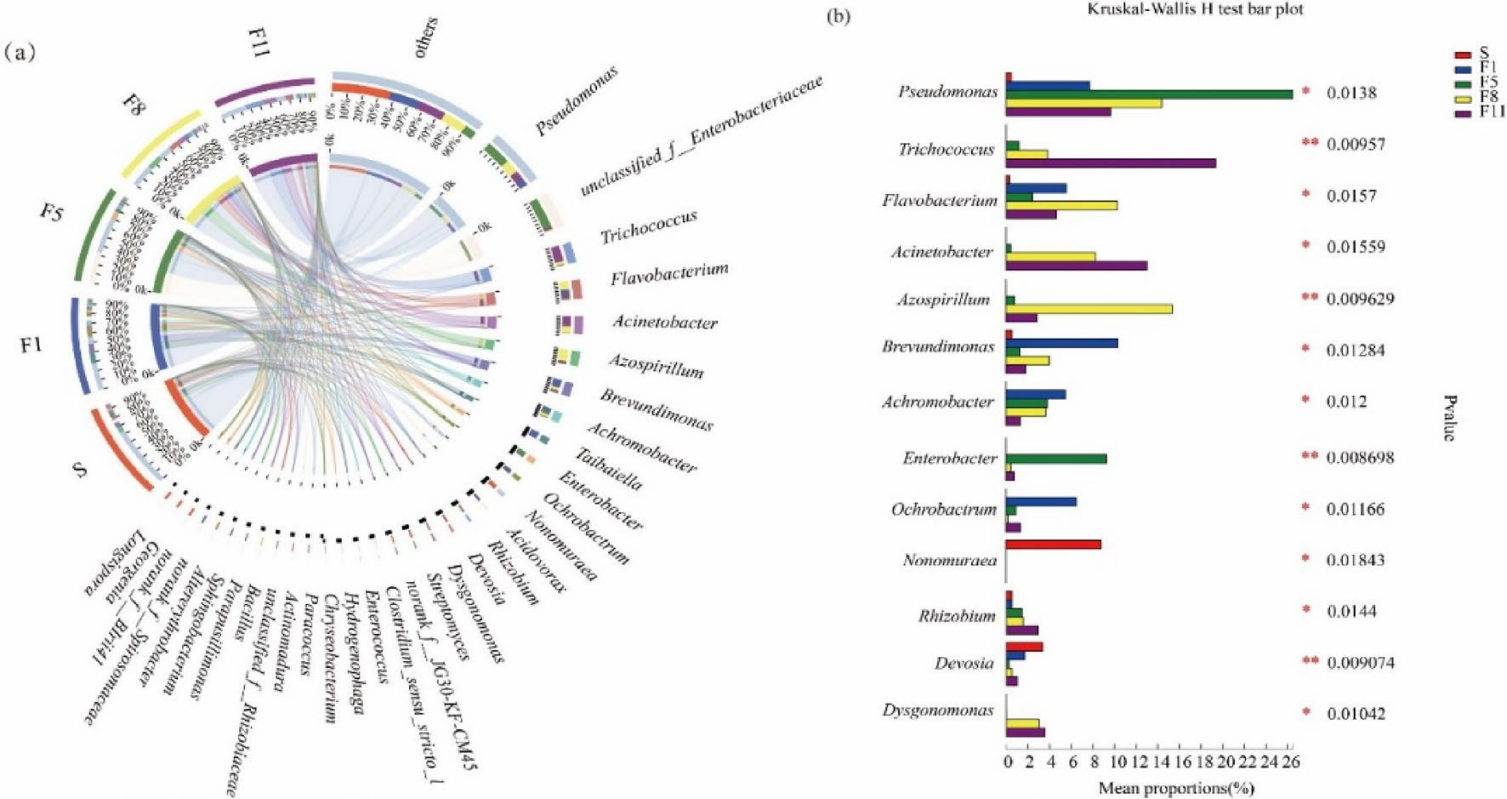
The (**a**) circos cluster analysis of dominant genera and (**b**) different analyses at the genus level.

At the genus level (Fig. 7), the relative abundance of *Pseudomonas* was 0.46% in the original sample(S), with its abundance was shown increased first and decreased then with culture time, reaching 8.75% in F11. The relative abundance of *Brevundimonas* was highest in the F1, was 10.79%, and which was significantly different from that in F5, F8 and F11. The relative abundance of *Flavobacteria* in the original sample(S) was 0.53%, which increased first and decreased then across culture stages. The relative abundance of *Devosia* was 3.09%, with its highest abundance at 1.71% in the F1 generation, after decreased with time. The relative abundances of *Achromobacter* and *Ochrobactrum* in F1 were 5.60% and 6.56%, respectively, but bacteria in these genera were rarely found in the original samples(S). Their relative abundances decreased with increasing culture time. In addition, *Trichococcus*, *Acinetobacter* and *Azospirillum* were found in F5, and the relative abundances of F11 were 19.65%, 13.01%, and 2.96%, respectively.

### Correlation analysis of microbial community at different culture stages

**Figure 8:**
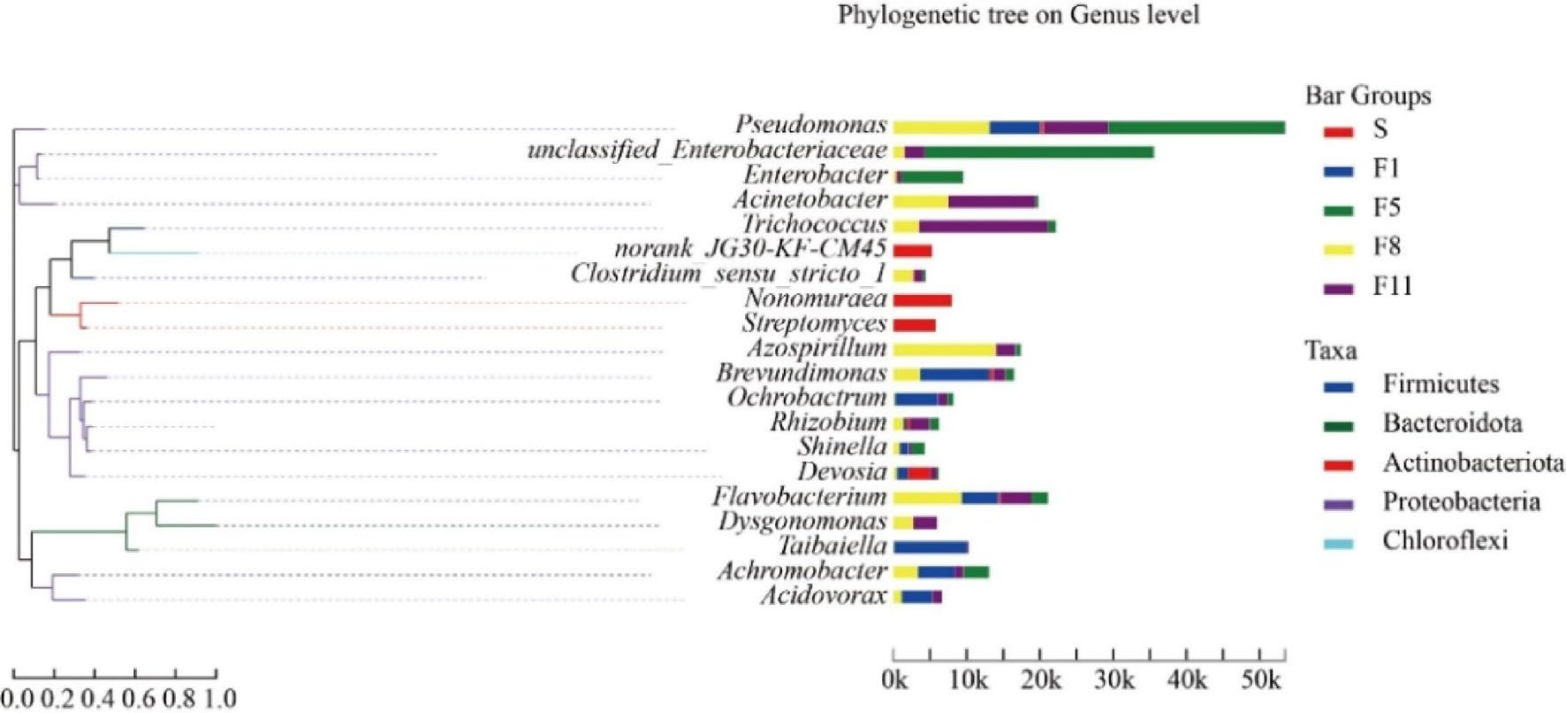
Microbial biological phylogenetic tree on the genus level.

The analysis of the dominant microbial community in the microbial consortium at different culture periods showed (Fig. 8) that Proteobacteria contained the largest number of genera with relatively distant evolution, and was mainly composed of 12 genera, including *Pseudomonas*, *Azospirillum*, *Brevundimonas* and *Ochrobactrum*. Among them, the abundance of *Pseudomonas* was dominant in the F1, F5, F8 and F11 generation samples, which played a key role in the degradation process of straw. Bacteroidetes was composed of *Flavobacterium*, *Dysgonomonas* and *Taibaiella* with similar evolution. It could be concluded that Proteobacteria was the main functional bacteria involved in straw degradation in samples of different culture periods.

**Figure 9:**
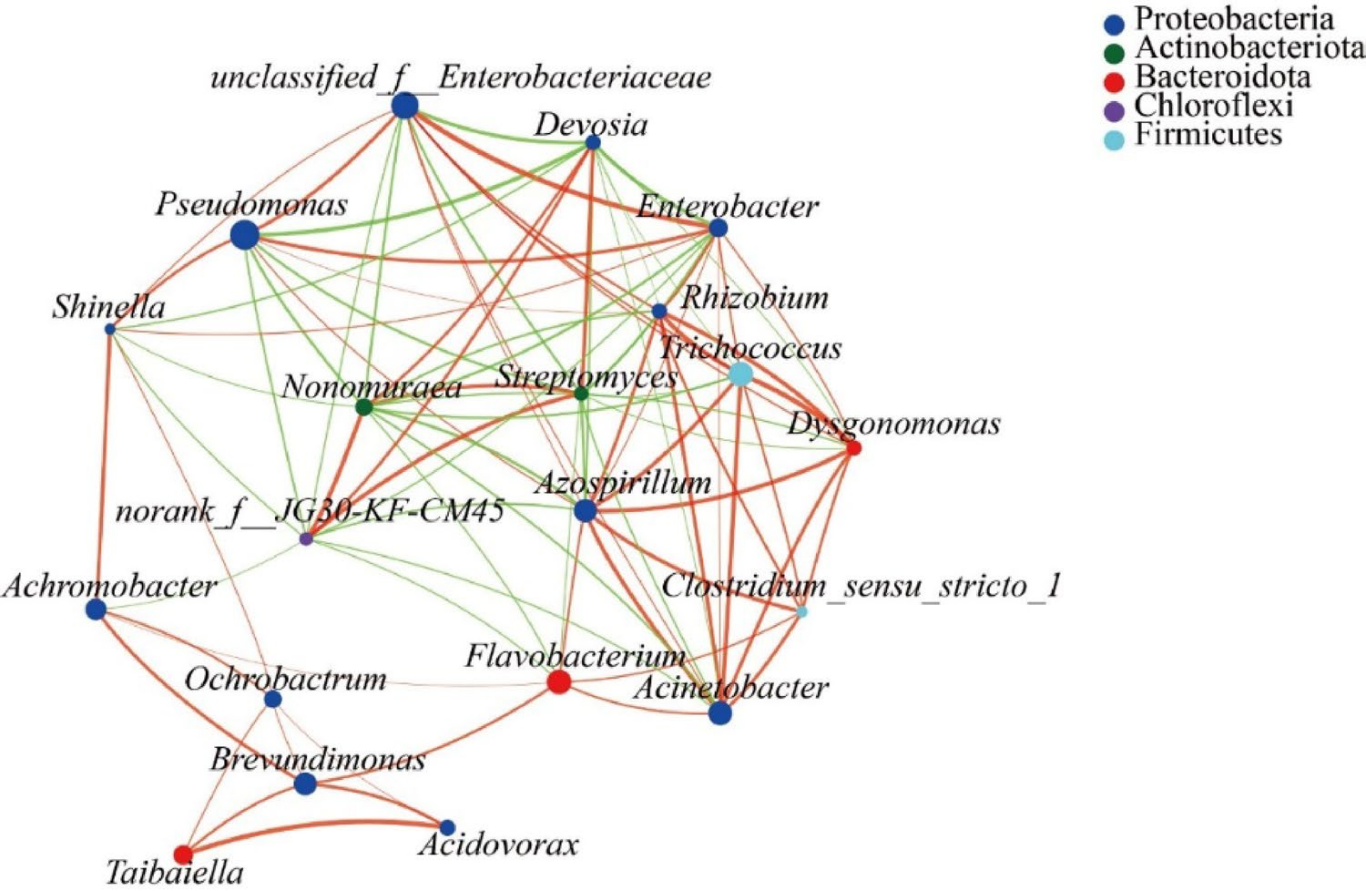
Correlation network diagram on genus level. The green line shows positive correlation, the red line shows negative correlation. The thickness of the line represents the size of the correlation coefficient.

The correlation network diagram was used to study the interrelationship between straw degrading microorganisms of M44 in different subculture periods (Fig. 9). *Rhizobium*, *Acinetobacter*, *Trichococcus*, *Dysgonomonas, Azospirillum*, *Enterobacter* were strongly correlated with each other and positively correlated with other bacteria. The results showed that there were significant interactions among different genera in the samples of different culture periods, and a variety of microorganisms synergistically degraded corn straw.

### Correlation analyses of physicochemical characteristics and dominant genera

**Figure 10:**
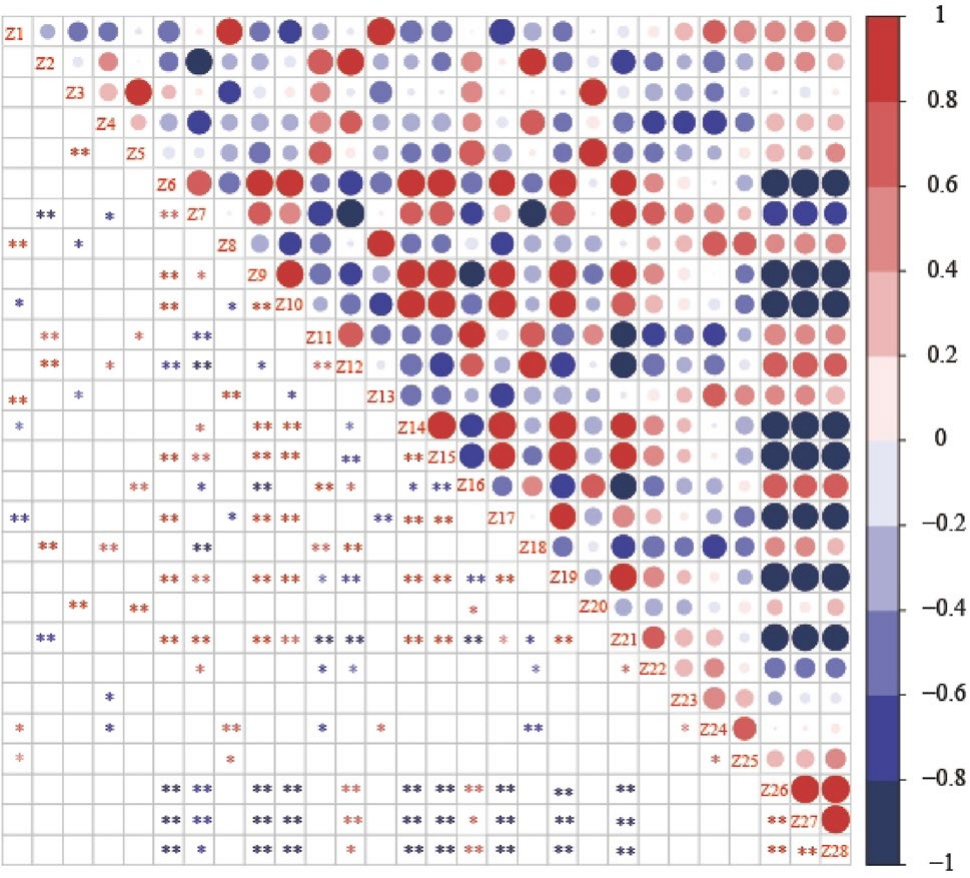
Correlation analysis of dominant genera and straw degradation characteristics at different culture stages. Z1: *Pseudomonas*, Z2: *Trichococcus*, Z3: *Flavobacterium*, Z4: *Acinetobacter*, Z5: *Azospirillum*, Z6: *Brevundimonas*, Z7: *Achromobacter*, Z8: *Enterobacter*, Z9: *Ochrobactrum*, Z10: *Acidovorax*, Z11: *Dysgonomonas*, Z12: *Rhizobium*, Z13: *Enterococcus*, Z14: *Hydrogenophaga*, Z15: *Chryseobacterium*, Z16: *Proteiniphilum*, Z17: *Devosia*, Z18: *Paracoccus*, Z19: *Sphingobacterium*, Z20: *Bacillus*, Z21: Endoglucanase, Z22: FPase, Z23: Xlyanase, Z24: Laccase, Z25: Lignin peroxidase, Z26:Acetic acid, Z27:Propanoic acid, Z28:Butyric acid. * and ** indicate significant correlations at the 0.05 and 0.01 levels.

Correlation analysis between the TOP20 genera in M44 and straw degradation characteristics (Fig. 10) showed that endoglucanase activity was positively correlated with *Brevundimonas*, *Achromobacter*, *Hydrogenophaga*, *Chryseobacterium*, *Sphingobacterium*, and some bacteria that degrade lignocellulosic or intermediate products in the colony. *Dysgonomonas* had a significant negative correlation with filter paper enzyme activity, *Acinetobacter* had a significant negative correlation with xylanase activity, and *Pseudomonas* and *Enterobacter* had a significant positive correlation with laccase and lignin peroxidase activity. *Rhizobium* and *Proteiniphilum* were positively correlated with acetic acid, prophetic acid, and butyric acid contents.

### Functional prediction analysis

#### The COG database comparison

**Figure 11:**
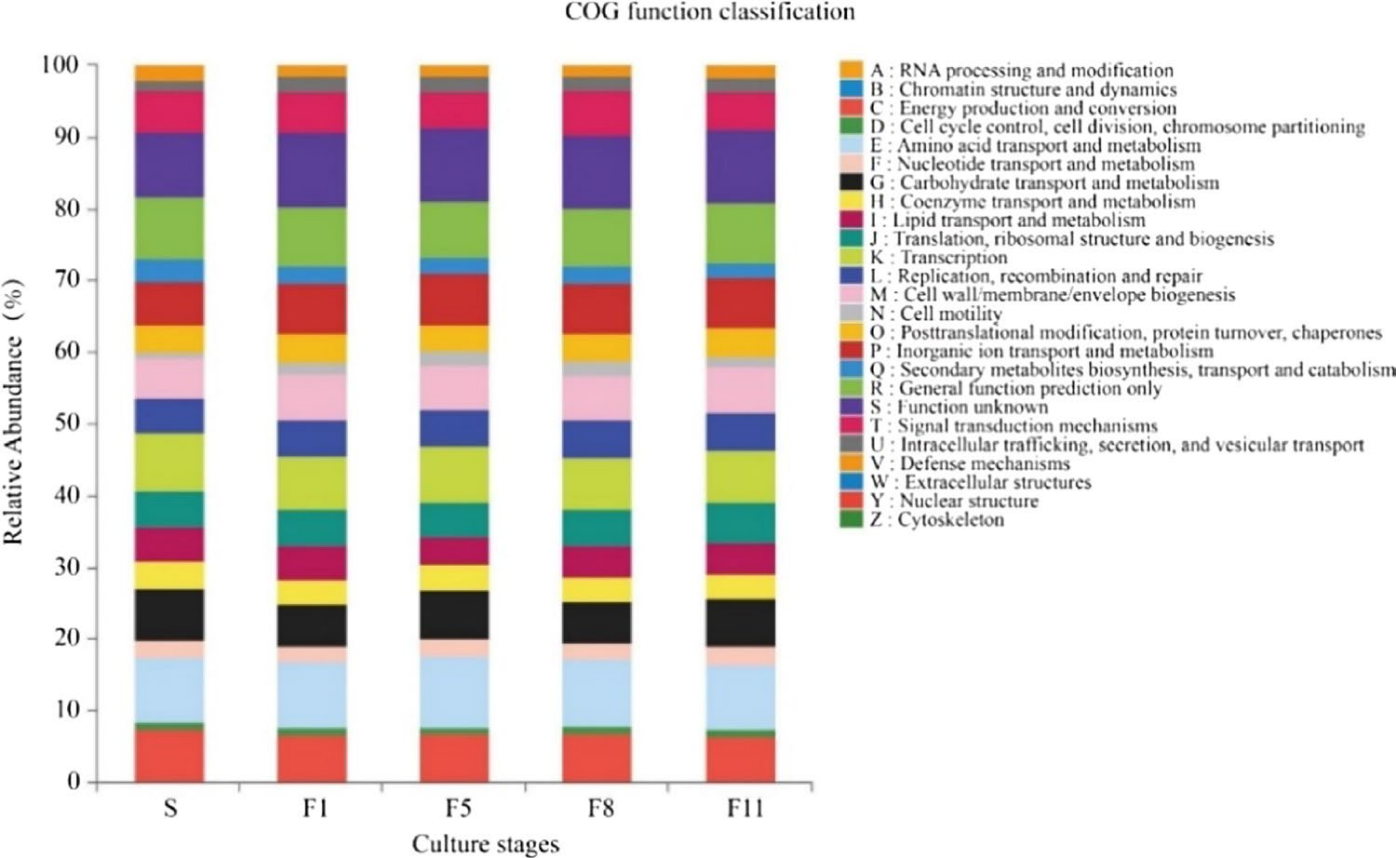
COG function classification at different culture stages.

Based on COG database comparison results (Fig. 11), it was found that the function of the M44 was mainly concentrated Amino acid transport and metabolism, General functional prediction only, Transcription, Carbohydrate transport and metabolism and Cell wall/membrane/envelope biogenesis and so on in different culture stages. It could be predicted that M44 may contain abundant genes related to protein decomposition, transport and metabolism enzymes, as well as a large number of genes related to cellulose and lignin degradation enzymes during subculture at low temperature.

#### The KEGG database comparison

According to KEGG level 1 (Table 1), genes in samples of different culture stages were mainly enriched in Metabolism, Environmental Information Processing, Genetic Information Processing, Cellular Processes, etc. Among them, Metabolism accounted for the highest proportion, and the proportion of original samples was 78.91%, which showed no significant difference with F1, F5, F8 and F11 generation samples. There were 46 metabolic pathways in KEGG Level 2. Table 2 showed the results of the top 15 pathway abundance values in different culture stages. The main metabolic pathways included Global and overview maps, Carbohydrate metabolism, Amino acid metabolism, Energy metabolism, Metabolism of cofactors and vitamins, Membrane transport and Signal transduction, etc. The top 30 enzymes in abundance were further analyzed (Table 3), the results showed that the relative abundance of DNA-directed DNA polymerase, DNA helicase, Peptidylprolyl isomerase, NADH:ubiquinone reductase (H( +)-translocating), 3-oxoacyl-[acyl-carrier-protein] reductase were high. In addition, the Peroxiredoxin, Acetyl-CoA carboxylase were present in different culture periods, and the abundance is obviously different.

**Table 1:** Abundance of metabolic pathways in KEGG level 1.

Pathway level 1	Relative abundance				
	S	F1	F5	F8	F11
Metabolism	71,804,522	68,396,950	69,357,496	68,222,337	66,077,491
Environmental information processing	5,217,082	5,523,127	8,007,265	6,281,961	6,063,911
Genetic information processing	5,555,747	5,531,476	5,096,113	5,482,298	5,793,948
Cellular processes	3,779,376	4,332,553	5,415,425	4,819,913	4,140,180
Human diseases	2,867,627	4,103,845	4,238,820	4,262,600	3,802,522
Organismal systems	1,768,482	1,826,120	1,687,914	1,771,249	1,670,516

**Table 2:** Abundance of main metabolic pathways in KEGG level 2.

Pathway level 2	Relative abundance				
	S	F1	F5	F8	F11
Global and overview maps	36,698,795	35,021,705	35,735,519	35,219,727	34,049,044
Carbohydrate metabolism	8,673,107	7,558,198	8,702,162	7,978,645	7,913,983
Amino acid metabolism	7,597,248	7,427,953	6,530,767	6,873,354	6,549,671
Energy metabolism	3,903,216	3,762,104	3,864,203	3,896,464	3,692,652
Metabolism of cofactors and vitamins	3,596,703	3,593,053	3,725,539	3,644,147	3,437,080
Membrane transport	3,095,153	2,889,293	4,447,515	3,345,208	3,489,770
Signal transduction	2,121,084	2,633,833	3,559,750	2,936,688	2,574,108
Cellular community—prokaryotes	2,174,308	2,191,543	2,868,655	2,456,078	2,299,346
Translation	2,226,147	2,217,846	1,952,172	2,197,749	2,362,025
Nucleotide metabolism	2,070,385	1,984,012	2,182,688	2,107,958	2,148,205
Replication and repair	2,075,040	2,131,660	1,947,021	2,067,261	2,188,123
Lipid metabolism	2,186,335	2,102,314	1,962,766	1,988,455	1,973,562
Xenobiotics biodegradation and metabolism	1,973,895	1,899,788	1,937,440	1,913,574	1,846,335
Metabolism of other amino acids	1,410,385	1,490,721	1,561,229	1,434,901	1,361,297
Biosynthesis of other secondary metabolites	1,509,132	1,382,209	1,173,759	1,245,861	1,187,638

**Table 3:** Abundance of main enzyme in KEGG database.

Enzyme	Description	Relative abundance				
		S	F1	F5	F8	F11
2.7.7.7	DNA-directed DNA polymerase	257,480.1	269,544.4	228,104.7	255,508.2	267,472
3.6.4.12	DNA helicase	239,467.7	246,707.9	224,557.6	238,236.4	261,575.2
2.7.13.3	Histidine kinase	203,894.1	198,846.7	217,314.4	215,296.2	224,476.4
1.6.5.3	NADH:ubiquinone reductase (H(+)-translocating)	229,278.6	240,233.9	129,918.5	182,624.6	168,860.6
5.2.1.8	Peptidylprolyl isomerase	133,037.1	192,070.5	188,614.8	170,111.8	176,382.4
1.1.1.100	3-Oxoacyl-[acyl-carrier-protein] reductase	158,389	124,941.8	91,544.37	118,301.1	107,359.8
1.9.3.1	Cytochrome-c oxidase	116,030.7	129,516.2	88,487.56	109,908.3	82,037.99
2.7.7.6	DNA-directed RNA polymerase	108,267	99,127.67	90,753.75	101,180	110,889
3.6.4.13	RNA helicase	66,236.02	104,128	134,162.7	103,132.5	97,727.01
2.5.1.18	Glutathione transferase	43,701.64	101,308.2	109,578.7	102,502.1	94,690.73
6.4.1.2	Acetyl-CoA carboxylase	90,429.95	83,013.37	70,743.19	78,453.64	86,584.13
6.3.5.7	Glutaminyl-tRNA synthase (glutamine-hydrolyzing)	97,086.25	81,673.35	55,880.64	81,853.13	85,283.83
6.3.5.6	Asparaginyl-tRNA synthase (glutamine-hydrolyzing)	96,945.24	81,040.35	55,870.64	81,663.14	85,197.51
2.2.1.6	Acetolactate synthase	85,634.95	77,886.08	91,255.27	74,534.3	70,764.46
2.3.1.9	Acetyl-CoA C-acetyltransferase	103,368.8	90,589.47	61,067.67	70,928.66	71,153.47
1.1.1.1	Alcohol dehydrogenase	71,673.16	56,966.11	89,150.36	78,660.21	79,576.49
3.6.3.14	H(+)-transporting two-sector ATPase	63,479.12	72,718.48	74,229.09	76,144.45	80,083.25
2.7.11.1	Non-specific serine/threonine protein kinase	136,970.2	61,816.57	49,102.02	61,017.16	54,550.24
3.6.3.34	Iron-chelate-transporting ATPase	97,434.59	51,016.2	85,437.47	67,417.31	61,494.66
4.2.1.17	Enoyl-CoA hydratase	88,389.35	75,819.41	67,554.28	68,716.31	56,118.12
3.6.3.17	Monosaccharide-transporting ATPase	111,557.7	42,082.87	89,111.98	54,910.08	58,474.91
3.6.1.27	Undecaprenyl-diphosphate phosphatase	72,599.74	63,598.02	63,013.71	75,157.35	74,602.76
3.4.16.4	Serine-type D-Ala-D-Ala carboxypeptidase	99,918.43	54,947.81	63,024.73	61,658.77	64,212.85
2.7.1.69	Protein-N(pi)-phosphohistidine–sugar phosphotransferase	43,858.59	32,919.81	111,785.1	57,650.64	93,291
1.2.4.1	Pyruvate dehydrogenase (acetyl-transferring)	89,505.4	64,665.76	48,704.92	70,471.22	60,318.52
1.17.4.1	Ribonucleoside-diphosphate reductase	64,268.07	70,183.88	70,697.2	63,249.07	64,438.91
4.2.99.18	DNA-(apurinic or apyrimidinic site) lyase	85,640.16	51,570.35	63,409.49	60,276.22	61,169.38
2.1.1.72	Site-specific DNA-methyltransferase (adenine-specific)	53,206	66,287.13	57,180.67	68,383.09	74,073.93
1.11.1.15	Peroxiredoxin	57,855.74	60,882.79	70,268.89	64,295.86	65,698.9
1.3.5.4	Fumarate reductase (quinol)	78,469.94	57,983.24	62,182.47	55,386.07	53,311.38

## Discussion

Many studies had shown that by simulating the decomposition process of lignocellulose under natural conditions (i.e., taking the original environmental samples as the inoculum and adopting restrictive culture techniques), composite flora that could efficiently degrade filter paper, rice straw, and pulp waste could be identified. In this study, the original material was taken from the dried dung sample of Hulunbuir city, Inner Mongolia, and mainly composed of Actinobacteria, Proteobacteria, Bacteroidetes, Firmicutes and Chloroflexi, which was rich in degraded cellulose, hemicellulose, lignin. The M44 was screened out from the original material by long-term restricted subculture, and the corn straw degradation rate was 35.90% at 15 °C for 21 days. The microbial community structure of the original samples was significantly different from that of the microbial consortia obtained after a long period of restricted subculture. The subculture process was not only a process of eliminating bacteria unrelated to straw degradation or not adapted to the medium conditions, but also a process of enriching lignocellulose-degrading bacteria, with randomness.

### Microbial diversity of microbial consortium with straw degradation

The M44 was a complex microbial that mix composed of aerobic bacteria, anaerobic bacteria, and strict anaerobic bacteria, and its microbiome structure changed considerably during low-temperature subculture. The Proteobacteria and Firmicutes were vital bacterial in different culture generation. As a reported, these types of bacteria were common in rice straw compost^20^, decaying wood^21^, and rumen^22^, which could produce laccase and degrade Kraft lignin^23^, and degrading lignin monoaryls, biaryls, and phenolic intermediates using extracellular laccases and peroxidases^24,25^. It is reported that *Clostridium* are anaerobic bacteria with a superior ability to decompose lignocellulosic materials and digest cellulosic waste in a methanogenic bioreactor^26–28^. However, it was not detected in this study, which may be due to the conditions of subculture and the unsuitable medium for its mass reproduction and growth.

*Acinetobacter*, *Azospirillum*, *Pseudomonas*, *Brevundimonas*, *Devosia*, *Achromobacter*, and *Chryseobacterium* played an important role in the straw degradation. Among them, *Acinetobacter* was found in cellulose-containing agricultural waste as the only carbon source, and efficiently secreted extracellular cellulase and hemicellulose enzymes^29,30^; *Azospirillum* had been shown to produce hydrogen peroxide enzymes, oxidase, methyl cellulase, and produced acetic acid, butyric acid, and lactic acid, and to participate in straw degradation metabolism^31,32^; Dye-decolonizing peroxidases (DYPs) secreted by *Pseudomonas* had the ability to degrade lignin and lignin model compounds^33,34^ and had high laccase and lignin peroxidase activities^35^. They were believed to be important functional bacteria for degradation of straw lignin. Studies had shown that *Brevundimonas* secreted oxidase and catalase to promote the decomposition of cellulose^36^; *Devosia* decomposed catalase and utilizes xylose, glyceraldehyde, cellulose, etc^37^; *Achromobacter* could oxidize xylose, secreted oxidase and xylanase, which effectively degraded cellulose and hemicellulose^38^; and *Chryseobacterium* decomposed cellulase and protease, degrading cell walls, and could cooperate with *Pseudomonas* to degrade cellulose and hemicellulose^39,40^, which correlated with xylanase activity. They were speculated to be functional bacterium for straw cellulose and hemicellulose degradation.

### Functional prediction of microbial consortium with straw degradation

The degradation of lignocellulose requires the joint action of a variety of enzymes produced by different microorganisms to attack the complex structure of its biomass^41^ and produce a fully complementary enzymatic system. Therefore, it is very important to explore some functional genes closely related to lignocellulose degradation. It was reported that the microorganism with high levels of carbon hydration gene had high degradation activity. Zhang et al.'s^42^ study showed that through the COG database compared, the gene for Carbohydrate transport and metabolism was the most; Singh et al^43^ found that Buffalo's rumen microbial group had a large number of functional genes for polysaccharide degradation. Based on COG and the KEGG database analysis, this study mainly was concentrated in Amino acid transport and metabolism, General functional prediction only, Carbohydrate transport and metabolism and Cell wall/membrane/envelope biogenesis, etc., which were similar to those of the above studies. In addition, samples with different culture generations in M44 contained Peroxiredoxin and Acetyl-CoA carboxylase, etc. Studies have shown that the Lig K enzyme and peroxidase could catalyze the degradation of lignin intermediates to pyruvate and oxaloacetate, which were finally degraded by tricarboxylic acid cycle^44,45^. In this study, it was speculated that corn straw lignocellulose could produce small molecular substances through different metabolic pathways under the action of transaminases, lyases and dehydrogenases, and finally completely degrade into organic acids or carbon dioxide.

## Conclusion

In nature, degradation of lignocellulose was coordinated by various active enzymes secreted by various microorganisms. In this study, *Pseudomonas*, *Azospirillum*, *Brevundimonas*, *Ochrobactrum* from Proteobacteria and *Flavobacterium*, *Dysgonomonas*, *Taibaiella* from Bacteroidetes, and *Devosia*, *Trichococcus*, *Acinetobacter*, *Rihizobium*, *Achromobacter*, *Chryseobacterium* were found to be the key bacteria for subculture progress. In different culture periods, the main metabolic pathways include Carbohydrate metabolism, Amino acid metabolism and Energy metabolism, etc. Furthermore, the M44 may contain a large amount of lignin biodegradable enzyme genes that could degrade the material, such as cellulose, hemicellulose and lignin. This study provided theoretical guidance for the selection of functional microorganisms of microbial consortium.

## Data Availability

The data used to analyze microbial diversity has been uploaded to the SRA database in NCBI with the entry number PRJNA777010.
